# Vestibular rehabilitation in multiple sclerosis: study protocol for a randomised controlled trial and cost-effectiveness analysis comparing customised with booklet based vestibular rehabilitation for vestibulopathy and a 12 month observational cohort study of the symptom reduction and recurrence rate following treatment for benign paroxysmal positional vertigo

**DOI:** 10.1186/s12883-020-01983-y

**Published:** 2020-11-27

**Authors:** J. Marsden, M. Pavlou, R. Dennett, A. Gibbon, R. Knight-Lozano, L. Jeu, C. Flavell, J. Freeman, D. E. Bamiou, C. Harris, A. Hawton, E. Goodwin, B. Jones, S. Creanor

**Affiliations:** 1 School of Health Professions, Faculty of Health: Medicine, Dentistry and Human Science, Peninsula Allied Health Centre, Derriford Rd, Derriford, Plymouth, PL6 8BH UK; 2 Academic Department of Physiotherapy, King’s College London, Room 3.5 Shepherd’s House, Guy’s Campus, London, SE1 1UL UK; 3 EAR Institute University College London, 332 Gray’s Inn Rd, London, WC1X 8EE UK; 4 Royal Eye Infirmary, Derriford Hospital, Plymouth, PL6 8DH UK; 5 School of Psychology, University of Plymouth, Drakes Circus, Plymouth, PL4 8AA UK; 6 Health Economics Group, University of Exeter, South Cloisters, St Luke’s Campus, Exeter, EX1 2LU UK; 7 Medical Statistics Group and Peninsula Clinical Trials Unit, Faculty of Health: Medicine, Dentistry and Human Science, Plymouth Science Park, 1 Davy Rd, Derriford, Plymouth, PL6 8BX UK

**Keywords:** Multiple sclerosis, Vestibular, Vertigo, Balance, Benign paroxysmal positional vertigo, Vestibulopathy, Rehabilitation, Randomised controlled trial

## Abstract

**Background:**

Symptoms arising from vestibular system dysfunction are observed in 49–59% of people with Multiple Sclerosis (MS). Symptoms may include vertigo, dizziness and/or imbalance. These impact on functional ability, contribute to falls and significant health and social care costs. In people with MS, vestibular dysfunction can be due to peripheral pathology that may include Benign Paroxysmal Positional Vertigo (BPPV), as well as central or combined pathology. Vestibular symptoms may be treated with vestibular rehabilitation (VR), and with repositioning manoeuvres in the case of BPPV. However, there is a paucity of evidence about the rate and degree of symptom recovery with VR for people with MS and vestibulopathy. In addition, given the multiplicity of symptoms and underpinning vestibular pathologies often seen in people with MS, a customised VR approach may be more clinically appropriate and cost effective than generic booklet-based approaches. Likewise, BPPV should be identified and treated appropriately.

**Methods/ design:**

People with MS and symptoms of vertigo, dizziness and/or imbalance will be screened for central and/or peripheral vestibulopathy and/or BPPV. Following consent, people with BPPV will be treated with re-positioning manoeuvres over 1–3 sessions and followed up at 6 and 12 months to assess for any re-occurrence of BPPV. People with central and/or peripheral vestibulopathy will be entered into a randomised controlled trial (RCT). Trial participants will be randomly allocated (1:1) to either a 12-week generic booklet-based home programme with telephone support or a 12-week VR programme consisting of customised treatment including 12 face-to-face sessions and a home exercise programme. Customised or booklet-based interventions will start 2 weeks after randomisation and all trial participants will be followed up 14 and 26 weeks from randomisation. The primary clinical outcome is the Dizziness Handicap Inventory at 26 weeks and the primary economic endpoint is quality-adjusted life-years. A range of secondary outcomes associated with vestibular function will be used.

**Discussion:**

If customised VR is demonstrated to be clinically and cost-effective compared to generic booklet-based VR this will inform practice guidelines and the development of training packages for therapists in the diagnosis and treatment of vestibulopathy in people with MS.

**Trial registration:**

ISRCTN Number: 27374299

Date of Registration 24/09/2018

Protocol Version 15 25/09/2019

**Supplementary Information:**

The online version contains supplementary material available at 10.1186/s12883-020-01983-y.

## Background

Vestibulopathy causes perceptual deficits (e.g. vertigo or dizziness, poor perception of vertical) and abnormalities in the control of eye movements and balance. Symptoms arising from vestibulopathy are common in people with Multiple Sclerosis (MS). Dizziness affects 49–59% of people with MS [1], with true rotational vertigo, an indicator of vestibular-induced dizziness, affecting approximately 20% [2]. In people with MS who report dizziness, 38.5% rate it as having a moderate or severe impact [1]. A greater severity of dizziness is associated with a lower quality of life [1]. Abnormalities in vestibular evoked ocular and spinal reflexes, that are important for the stabilisation of gaze and balance, are seen in 40–86% of people with MS. [3–9] Vestibulopathy, combined with clinical signs of lower limb weakness, sensory loss, ataxia and spasticity, may result in balance and mobility impairment [10–13]. This can result in falls and injuries, restriction in outdoor mobility and a subsequent impact on social participation and quality of life for individuals [14–19]. The balance dysfunction and reduced mobility seen in people with MS are further associated with significant health and social care costs [18, 20].

The vestibular system consists of a peripheral pathway (the inner ear and vestibular nerve) and central pathways in the brain (e.g. the vestibular nuclei and cerebellum) that process vestibular signals [21]. Although MS affects the central nervous system (CNS), people with MS can present with peripheral symptoms if the lesion affects the vestibular nerve in isolation as it enters the CNS [22]. Further, Benign Paroxysmal Positional Vertigo (BPPV), a condition affecting the inner ear, has been reported in around 50% of people with MS who attend specialised neuro-otology clinics [22, 23]. The cause and management of BPPV is different to that of peripheral or central vestibulopathy, hence it is important to identify this condition in order to manage it appropriately.

In BPPV, otoconia crystals become dislodged from the otolith macula and become trapped within the semicircular canals; usually the posterior canal is affected. BPPV can be diagnosed using the Dix-Hallpike manoeuvre that moves the head in the plane of the posterior canal. When otoconia are present in the canal this leads to deviation or deflection of the cuplua and reflexive, characteristic eye movements. BPPV is treatable with bedside physical manoeuvres such as Epley or Semont, which aim to move these crystals back into the otoliths. A Cochrane systematic review highlights that compared to no intervention these manoeuvres, in otherwise healthy participants, are very effective at reducing symptoms of vertigo (odds ratio 4.42[2.62–7.44]) and producing a negative Dix –Hallpike test (odds ratio 9.62 [6–15.42]) [24]. However, symptoms can re-occur in 36% of cases over 48 months [24] and re-occurrence rates are greater in people with migraine or head injury [25, 26]. It is unclear whether repositioning manoeuvres have the same short and long term effectiveness in people with MS compared to the general, otherwise healthy, population. Currently, testing and repositioning manoeuvres for BPPV in people with MS are not routinely performed. Therefore, nested within this study, we plan to undertake a observational study to determine the success rate of repositioning manoeuvres and re-occurrence rate of symptoms in pwMS who are assessed as having BPPV.

Prognosis for recovery with rehabilitation for vestibulopathy in the general population varies with aetiology, being greater for peripheral disorders compared to central disorders [27]. Symptom recovery involves adaptive changes in the brain, termed *vestibular compensation* [27]. Recovery can be affected by other factors such as additional sensory dysfunction (somatosensory and/or visual), restricted head motion, lack of mobility, long term use of anti-vertiginous drugs, fatigue and psychological problems such as depression, phobias and anxiety [28]. Therefore, identifying factors affecting prognosis may, in future, aid screening and management of people with MS and vestibulopathy. Clinical tests such as HINTS (head impulse, nystagmus and skew deviation) can differentiate peripheral and central vestibulopathy [29]. If these tests can be shown to be as sensitive and specific as laboratory-based measures (e.g. using a rotary chair and videonystamography) in people with MS, the use of such tests would aid in the clinical diagnosis and planning of treatment.

Vestibular rehabilitation (VR) is the standard of care for people with vestibulopathy. VR involves progressive exercises including eye, head, and body movements in sitting, standing, and walking. In otherwise healthy people with a peripheral vestibular disorder, a Cochrane systematic review concluded that there is moderate to strong evidence to support VR as a safe, effective management option, with clinically and statistically significant improvements noted for perceptual, oculomotor and balance symptoms [30]. In contrast, the effectiveness of rehabilitation for dizziness where solely central vestibular pathways have been damaged has only been explored in studies with small numbers of patients with varying pathology [31–37]. These studies suggest that improvements in symptoms and balance for people with a central vestibular disorder can be achieved, although not always to the same extent as in people with peripheral vestibular disorders. However, these studies did not include people with MS. Rather, the studies involve people with hereditary conditions, head injury, or stroke. The studies were retrospective or isolated case reports and did not have concurrent control groups. Furthermore, the studies frequently did not use validated outcome measures.

Two recent systematic reviews have investigated the effectiveness of VR in people with MS. [38, 39] Synnott and Baker reviewed the effect of VR reported in seven RCTs [40–46] representing a total of 323 people, aged 20–63 with a range of MS phenotypes [38]. The authors reported a wide variety of VR treatment protocols with respect to content and intensity. Frequency of supervised VR sessions varied from twice daily to once a week, with four studies incorporating a home exercise programme between 1 and 7 days a week. Similarly, the duration of interventions were variable ranging from 4 to 14 weeks. Four studies compared VR with usual care, two with neurological rehabilitation, and one compared VR customised to the participants’ symptoms with a standard format VR. All included studies investigated the effectiveness of VR on a balance related outcome measure. In addition, three studies investigated the effectiveness of VR on dizziness, and three on fatigue. These authors concluded that VR is a safe and effective intervention offering short term improvements in balance in people with MS. However, they also noted that evidence for optimal VR prescription and long-term effects of VR is limited. The second review [39] included seven articles [40, 41, 44–48], reporting on six RCTs of VR in people with MS (*n* = 321, mean age 43.6 years). Two of the articles were different to those included in the previous review [47, 48]. The authors concluded that VR is more effective than no intervention for gaining improvements in balance, dizziness and fatigue in people with MS. However, they reported a non-statistically significant difference between groups when VR was compared with other exercise interventions, suggesting that there is insufficient evidence to conclude that VR is more effective than other exercise-based interventions.

To date, only one pilot study has evaluated a customised approach to VR compared to generic VR in people with MS [42], and the cost-effectiveness of such interventions has not been explored in people with MS. Customised VR programmes include a comprehensive assessment of balance and oculomotor control and the provision of exercises specifically selected to treat each individual’s identified impairment or functional limitations and address their individual goals. In otherwise healthy people with peripheral vestibulopathy a customised, individualised VR programme that targets patient-specific problems has been demonstrated to be more effective than a generic VR exercise programme [49–51].

Many people with vestibular disorders also report symptoms of visually induced dizziness, which refers to symptoms specifically triggered or exacerbated by complex, unusual or moving visual stimuli, including crowds, scrolling on the computer screen and watching moving traffic [52]. It is a frequent and at times debilitating symptom associated with high levels of disability, prolonged illness and poorer clinical outcome in people with vestibulopathy [53]. Visually induced dizziness responds well to customized VR programmes that incorporate structured exposure to visual motion stimuli [54].

Despite this evidence, standard care of isolated vestibular symptoms in otherwise healthy participants in many UK centres continues to include, at best, only standardised, generic exercises, delivered using a booklet after an initial one-to-one session or group class. Given the complexity of symptom presentation in people with MS it may be that customised exercises are more effective and cost effective than home based generic exercises delivered via a booklet.

## Aims

The primary aim of this research is to compare the clinical and cost effectiveness of a 12-week VR programme, consisting of 12 face-to-face sessions and a customised home-based programme plus usual care, to a 12-week generic booklet-based home programme with telephone support plus usual care, in ambulant people with MS with associated peripheral and/ or central vestibulopathy.

The secondary aims are to investigate factors affecting recovery from vestibulopathy with VR, and to explore the sensitivity and specificity of clinical bedside tests in diagnosis of central and peripheral vestibulopathy and in predicting treatment outcome. The nested observational study will determine the success and reoccurrence rate of symptoms with repositioning manoeuvres in pwMS who have clinically defined BPPV.

## Objectives

1. Assess the clinical effectiveness of a customised VR programme compared to a generic booklet-based VR programme on subjective reports of the perceived impact of vertigo or dizziness symptoms as measured by the Dizziness Handicap Inventory (primary outcome).
2. Assess the clinical effectiveness of a customised VR programme compared to a generic booklet-based VR programme on self-reported balance confidence and walking ability and objective measures of standing balance, functional gait and visual dependency for perceptual orientation responses (secondary outcomes).
3. Assess the adherence to a customised VR programme compared to a generic booklet-based VR programme.
4. Establish the intervention costs of the customised and generic booklet-based VR and conduct a full cost-effectiveness analysis.
5. Explore the impact of the underlying vestibular pathology (peripheral, central, or combined) and associated symptoms (weakness, distal somatosensory loss, visual dependency, psychological state) with treatment outcome.
6. Explore the sensitivity and specificity of bedside tests for the diagnosis of central and peripheral vestibulopathy and in predicting treatment outcome.
7. To determine the success and reoccurrence rate of symptoms with repositioning manoeuvres in pwMS who have clinically defined BPPV.

## Methods

### Randomised controlled trial design

The study is a multi-centre parallel group, superiority randomised controlled trial (RCT) with blinded outcome assessment. Ambulant individuals with MS and peripheral or central vestibulopathy will be randomised in a 1:1 ratio to either a customised 12-week VR programme consisting of 12 face-to-face sessions and a customised home-based programme plus usual care (“Intervention group”), or a generic home-based exercise programme delivered using a booklet and telephone support plus usual care (“Control group”).

### Observational study design

A longitudinal cohort observational study will determine the success and re-occurrence rate of symptoms with repositioning manoeuvres in pwMS who have clinically defined BPPV.

### Trial settings

The sites involved are based in two geographical regions of the UK: South West England (Devon and Cornwall) and London.

## Participants

### Eligibility criteria

#### Inclusion criteria

The study population will comprise individuals diagnosed with MS (relapsing remitting, primary or secondary progressive) according to McDonald’s revised criteria [55, 56]. These participants will:

- be aged > 18 years
- be willing and able to consent
- score 1–6 on Patient Determined Disease Steps, equivalent to Expanded Disability Status Scale (EDSS) 2–6.5
- report one of the following at least 4 times/month:
  - ◦ feeling that things are spinning or moving around
  - ◦ a feeling of being light-headed, “swimmy” or giddy
  - ◦ feeling unsteady and about to lose balance
- willing and able to travel to, and participate in, the 12 face to face sessions should they be allocated to the intervention group and to commit to undertaking their individualised home-based programme
- willing and able to travel to local assessment centres for screening and baseline tests and blinded outcomes assessment.
- People eligible for the nested observational study will in addition have a a positive diagnosis of BPPV determined by tests for posterior (Dix- Hallpike) and horizontal canal BPPV (horizontal head rotation in supine with the head in 30^o^ flexion) [57].

#### Exclusion criteria

People will be excluded if they:

- have neurological conditions other than MS as determined from clinical notes
- have relapsed or received steroid treatment within the last month
- currently or recently (within past 6 months) participated in a VR programme
- have an orthopaedic deficit which may impact on postural and gait testing or significant pain or weakness (> 4/10 on a numerical rating scale) associated with osteo- or rheumatoid arthritis
- have dizziness solely explained by other causes (e.g. postural hypotension)
- have a headache or migraine associated with a subjective report of one of the following at least 4 times/month:
  - ◦ nausea (feeling sick), stomach churning
  - ◦ vomiting
- have been taking vestibular sedatives specifically for the treatment of vertigo for more than 4 weeks. If people have been regularly taking vestibular sedatives (> 4 weeks) and, with approval of their neurologist and/or GP, they stop the medication then they will be eligible to take part in the study after a 6 week wash out period following re-screening.

Identification and recruitment of participants will be via a number of routes, including screening regional MS databases, healthcare professionals, and advertising via local MS support groups and newsletters.

### Sample size

#### Randomised control trial

The primary outcome measure is the Dizziness Handicap Inventory (DHI) [58], assessed at the primary endpoint of 26 weeks (±2 weeks) post randomisation. The sample size calculation is based on data from a MS vestibular waitlist control study [40], where the mean between-group differences in the change in DHI were 16.5 units and 18.1 units for the intervention vs exercise group and intervention vs wait-listed group, respectively; equivalent to standardised effect sizes (for change in DHI) of 1.03 and 1.12. The study reported a standard deviation of the difference between DHI at baseline and end of intervention of 20.7 for the intervention group and 9.6 for the wait-listed control group. Pooling these values gives an estimated standard deviation of the changes in DHI of 15.9, which was used in the sample size calculation for this trial.

Primary outcome data (change in DHI) based on 25 participants per group would enable a standardised effect size of ~ 0.94 to be detected with 90% power, or 0.81 with 80% power, at the two-sided 5% significance level. To account for participant drop out, the aim is to recruit at least 70 participants (35 per allocated group).

#### Observational study

Based on being able to recruit 140 participants to the main project (given the resources available), and assuming approximately 50% of pwMS present with BPPV [22, 23], it is anticipated that approximately 70 people will be enrolled in the observational study. The primary aim of the observational study is to estimate the recurrence rate of BPPV. Based on data from Hilton et al. 2014 [24], the expected precision of the estimate of recurrence, based on a range of sample sizes and recurrence rates, and after allowing for 10% loss to follow-up by the end of the one-year period, are provided in Table 1.

**Table 1:** Expected precision of the estimate of recurrence, based on a range of sample sizes and recurrence rates, after allowing for 10% loss to follow-up by the end of the one- year period. These are based on exact confidence intervals for the proportion of recurrences, using the Clopper-Pearson method

Sample size		Estimated margin of error (%)					
Total number of participants recruited to trial 2	Total number of trial 2 participants followed-up	Recurrence rate (%)					
		10	15	20	25	30	35
30	27	13.4	14.8	15.9	17.6	18.2	18.7
35	32	11.5	13.8	14.6	16.0	16.5	17.3
40	36	11.5	12.4	13.9	15.0	15.9	16.5
45	41	10.2	11.8	13.0	14.0	14.7	15.3
50	45	10.2	11.5	12.5	13.3	14.2	14.7
55	50	9.2	10.5	11.8	12.6	13.4	13.8
60	54	8.6	10.3	11.4	12.3	12.8	13.3
65	59	8.5	9.9	10.9	11.7	12.3	12.7
70	63	8.0	9.3	10.6	11.3	11.9	12.3
75	68	7.9	9.1	10.2	10.8	11.4	11.9
80	72	7.5	8.9	9.7	10.5	11.1	11.5

### Screening

**Fig. 1:**
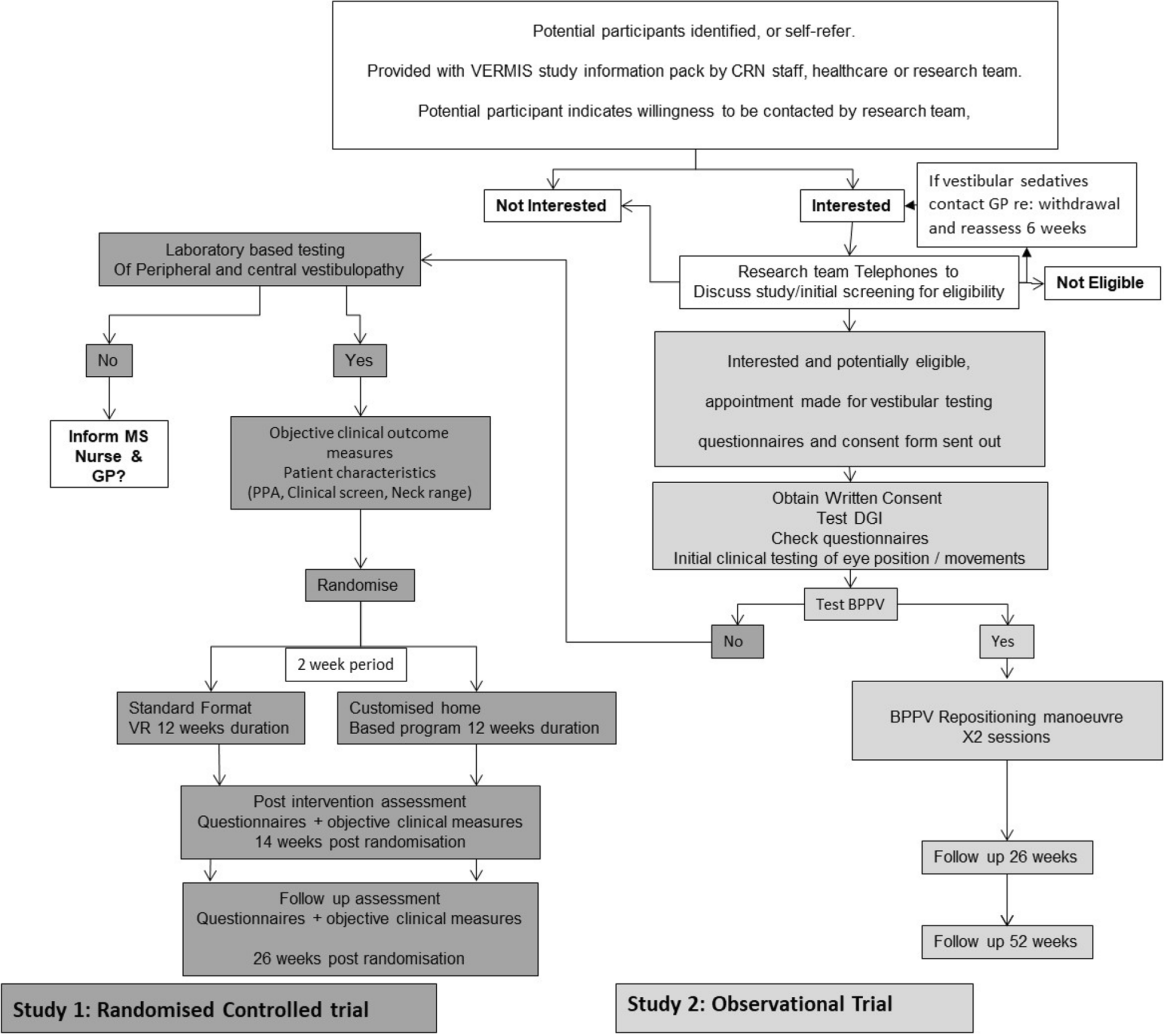
Flow diagram of patient pathway

Figure 1 indicates the flow of participants. Initially, people will be screened by telephone for subjective symptoms of vertigo and dizziness and poor balance, based on questions 4, 6 and 10 of the Vertigo Symptom Scale (short version [59]).

If deemed eligible following telephone screening, they will attend an initial first face-to-face visit.

#### Screening for BPPV

Participants will be screened for posterior canal BPPV using the Dix-Hallpike manoeuvre and horizontal canal BPPV using a roll test. Those with a positive BPPV test will be excluded from the RCT and treated with the appropriate re-positioning manoeuvre. After providing informed consent, these individuals will then be entered into an observational study and followed up at 6 and 12 months to assess for any re-occurrence of BPPV, as detailed in the section “Nested observational study”.

#### Screening tests for peripheral and central vestibulopathy

People who are negative for BPPV will then be screened for signs of peripheral and central vestibulopathy using a neuro-otological assessment. This will be conducted by a Band 7 Audiologist with support of the Research Therapist at the Royal Ear, Nose and Throat Hospital London and the trained Research Therapist at the Plymouth University site with support from a consultant clinical scientist. The assessment will include a videonystagmography (VNG) recording of:

- gaze (+/− 30°) with/without optic fixation,
- saccades (at 0° and +/− 30°, assessing for velocity, accuracy, main sequence and disconjugacy (internuclear ophthalmoplegia INO),
- smooth pursuit at 0.2, 0.3 and 0.4 Hz (with peak velocities of 38, 56.5, and 76°/s, respectively, assessing for saccadic intrusions),
- optokinetic responses to a full-field striped display moving at 40°/s (assessing for symmetry),
- Vestibulo-ocular reflex (VOR): Sinusoidal rotation at 0.2 Hz with/without visual fixation and impulsive rotation (until nystagmus subsides, approximately 45 s - maximum 100 s later) with an initial 140°/s acceleration/deceleration and a 60°/s fixed-chair velocity
- VOR suppression. VOR suppression is considered normal when no measurable nystagmus was recorded during visual fixation.

Based on the pattern of responses the participants will be classified as having:

- No vestibular pathology and will not be included in the study
- Peripheral unilateral vestibular impairment
- Peripheral bilateral vestibular hypofunction
- Central vestibular impairment
- Combined central and peripheral vestibulopathy.

### Randomised control trial

#### Randomisation

Participants identified to have a peripheral or central vestibular impairment will be invited to participate in the RCT. An online web-based system (www.redcapcloud.com) provided by the UKCRC-registered Peninsula Clinical Trials Unit (PenCTU) will be employed for randomisation. A minimisation procedure with a random element will be used to allocate participants to either the generic booklet-based programme (control group) or the customised VR Programme (intervention group). The following factors will be used in the minimisation procedure:

- Diagnosis: Peripheral (unilateral or bilateral) vs central/ combined vestibulopathy
- Severity of dizziness: DHI ≥59 or DHI < 59. People with MS with a score of over 59 have higher rates of falling [60]
- Fampridine: Prescribed or not prescribed
- Region: South West England or London

#### Blinding

Participants will be not be blinded to group allocation as the intervention arm involves weekly one-to-one sessions with the Treating Therapist at the respective study site. Following randomisation via the online web-based system (www.redcapcloud.com) the treating therapist is informed via e mail of the group allocation. The Treating Therapists and health care providers are also unable to be blinded due to the nature of the programmes. However, the Research Therapists undertaking the outcome assessments will be blinded to the participants’ allocated group.

The initial baseline assessment will be undertaken prior to randomisation ensuring these assessments are blinded. Every effort will be made throughout to ensure that subsequent assessments are blinded, for example by reminding participants at the start of the visit not to discuss their exercises or physiotherapy with the blinded Research Therapist. At each assessment time point, the blinded Research Therapists will be asked to record on the case report form (CRF) whether or not they have been un-blinded to group allocation, and if so the reason for this. In cases where Research Therapists were not un-blinded they will be asked to guess the group allocation for each participant.

### Interventions: randomised controlled trial

#### Generic booklet-based VR (control group)

The generic VR exercise group will undertake a one-hour individualised physiotherapy session during which they will receive the validated self-management booklet ‘Balance Rehabilitation’ [61]. The booklet explains how VR exercises help to improve vestibular symptoms and provides instructions on how to conduct the exercises. The Treating Therapist will provide further verbal instructions on how to use the booklet. Participants will be asked to practise the exercises unsupervised at home for 10 min, twice a day for 12 weeks and to fill out a daily diary sheet indicating treatment duration and content. They will receive telephone support from the Treating Therapist in the form of two 15 min phone calls, one in week 1 and one in week 4. These contacts will be guided by an interview schedule and focus on adherence, barriers to adherence and discussion of any concerns and queries regarding the exercise programme.

#### Customised VR (intervention group)

The customized vestibular rehabilitation group will receive 12 individualised, 1 h, vestibular rehabilitation sessions over a 12 week period, typically on a weekly basis. Sessions will be supervised by the Treating Therapist. Each participant will practice a selection from the following type of exercises:

- Eye, head, and postural exercises that provoke a patient’s symptoms
- Gaze stabilization exercises
- Exercises to re-train postural alignment and movement strategies
- Re-training sensory strategies
- Learning to adapt postural strategies to changing contexts
- Dual task training while walking
- Postural orientation exercises
- Neuromuscular (ankle-hip-stepping motor strategies) postural strategies.

The exercises will be determined following an initial clinical assessment of oculomotor function, balance and mobility based on subjective report and objective tests of the VOR and eye-head coordination, the mini balance evaluation system test (mini-BEST test) [62], motion sensitivity quotient test [63], the gait assessment and intervention tool (G.A.I.T) [64] and a visual assessment of postural alignment. The Treating Therapist will select exercises in partnership with the participant during the supervised session. Progress in specific areas will be objectively assessed at each supervised session, any concerns will be discussed, exercises that have not yet been included in the home programme added and practised, and existing exercises modified to gradually increase task difficulty. Each participant will be provided with an individualised home exercise programme of 3–5 exercises to practise each for 1-min, twice daily on days they do not have a session with the therapist. This will include video links to a demonstration of the exercise and progression rules. For those with any reported symptoms of migraine, at first, three exercises only will be provided but progressed to five exercises if no noticeable exacerbation of symptoms beyond the exercise period is noted. Exercises will target vestibular-related symptoms; for example, if VOR is affected, gaze stabilisation training will always be included. One out of the five exercises can target other systems deemed relevant (by the Treating Therapist or participant) to balance and mobility (e.g. mobilisation or stretches for reduced neck motion). At the last supervised session, a home maintenance exercise programme will be provided.

#### Adherence and standardisation / fidelity to the interventions

Both groups of participants will be asked to complete a daily diary to record the duration of home exercise practice. This data will be reported as percentage completion of prescribed exercises. In addition, adherence in the customised group will be reported as the number of face-to-face sessions the participant attended.

The Treating Therapists based at the two sites will be responsible for delivering the interventions. Therapists will attend a 2 day training course at the start of the trial which will cover the theory and practice of vestibular rehabilitation, trial methods and the importance of adhering to protocol. The Treating Therapists will complete a standardised proforma outlining the exercises taught in both groups, the agreed frequency of training as well as advice provided in the one-to-one or telephone follow ups. This will enable assessment of intervention fidelity and potential contamination effects.

#### Data collection and outcome measures

Participant characteristics including demographics, type of MS, medication and co-morbidities will be documented at baseline using a customised CRF. Participants will additionally have a clinical screen for vestibulopathy [29, 65, 66]. This includes HINTS (the head impulse sign, presence and type of nystagmus and skew deviation) and truncal ataxia [29, 65, 66]. Potential factors affecting balance and mobility [67**],** including objective measures of isometric knee extensor strength (assessed in sitting using a strain gauge), distal leg sensation (as determined by 10 g monofilaments), reaction time (using an online program https://faculty.washington.edu/chudler/java/redgreen.html) and neck passive range of motion (using goniometry), will also be assessed.

Standardised and validated clinician-rated assessments and patient self-reported clinical outcomes will be measured at baseline prior to randomisation (T0). Baseline measurements and randomisation (T0) occurs 2 weeks prior to the participant commencing an intervention. Measures will also be taken post-intervention at 14 weeks (+/− 2 weeks) post-randomisation (T14) and 26 weeks (+/− 2 weeks) post-randomisation (T26).

#### Primary outcome measure

The primary outcome measure is the impact of dizziness on daily function assessed using the DHI at the primary endpoint of 26 weeks (+/− 2 weeks) post-randomisation, and will also be collected at the secondary endpoint of 14 weeks (+/− 2 weeks) post-randomisation [58]. The DHI is a validated 25-item self-report questionnaire that assesses three domains: functional, emotional and physical. Responses to each question are graded 0 (no), 2 (sometimes) or 4 (yes). The scores per section are summed to give a maximum score of 100 points. Higher scores indicate a greater perceived impact of dizziness. The DHI is reliable in people with MS (intraclass correlation coefficient = 0.90, 95% confidence limits 0.77–0.96) [68].

The primary economic endpoint is the quality-adjusted life-year (QALY) assessed using the EQ-5D-5L [69]. QALY weights will be derived using the ‘cross-walk’ [70] to the EQ-5D-3L UK tariff [71], as recommended by the National Institute for Health and Care Excellence (NICE) [72].

#### Secondary outcome measures

The following outcomes are assessed at both T14 and T26:

- Functional ambulation using the Dynamic Gait Index [68, 73–75]
- Static and dynamic visual acuity assessed using an Early Treatment Diabetic Retinopathy Study (EDTRS) chart with and without passive head motion (40^0^ yaw rotation at 1.5 Hz) [76, 77]
- Visual dependency assessed using the Rod and Disc test [78, 79]
- Cognitive impairment and processing speed assessed using the Symbol Digit Modalities Test (SDMT) [80]
- Impact of MS on walking assessed using the 12 item self-report walking scale (MSWS-12) Version 2.0 [81]
- Perceived confidence in performing activities of daily living assessed using the self-report Activities-Specific Balance Confidence Scale [82]
- Self-reported symptoms of poor balance and increased anxiety and arousal assessed using the Vertigo Symptom Scale- Short Form [59, 83]
- Symptoms of visually induced dizziness symptoms assessed using the self-report Situational Characteristic Questionnaire [52]
- Fatigue as assessed using the self-report Fatigue Scale for Motor and Cognitive functions (FSMC) [84]
- Symptoms of depression and anxiety assessed using the self-report Hospital Anxiety and Depression Scale (HADS) [85]
- Health-related quality of life using the 29-item Multiple Sclerosis Impact Scale (MSIS-29) Version 2.0 [86], a disease specific patient-reported outcome measure [87]
- QALYs assessed by the Multiple Sclerosis Impact Scale – eight dimensions (MSIS-8D) and the Multiple Sclerosis Impact Scale – Eight Dimensions Patient version (MSIS-8D-P) [88, 89]. Both measures are based on responses to the MSIS-29, and will be used in sensitivity analyses
- Retrospective diary of falls over the past month for baseline measure and prospective daily falls diary over 12 weeks for assessment 2 (T14) and follow up (T26)
- Treatment adherence as determined by the use of patient reported diaries and reported as the percentage of completed prescribed exercises and attendance at face to face sessions
- User experience of the intervention will be explored through brief semi-structured face to face individual exit interviews based on an interview schedule.

### Safety monitoring

Participants will be monitored for adverse events via completion of adverse events forms, during telephone or face-to-face contacts as part of the intervention phase and during follow-up assessments. New or worsening problems which participants perceive to be related to participation in VR, as well as any relapses and falls, will be reported. If a member of the research team becomes aware of a severe adverse event, they will report this to the Chief Investigator within 24 h, and the trial sponsor will be informed. The Trial Management Group and the Trial Steering Committee will be informed of the details of all adverse events. There are no special compensation arrangements for harm arising from the study although neglectful harm will be covered by the insurance scheme of the sponsor organisation.

### Statistical analysis

A detailed statistical analysis plan (SAP) will be developed during the delivery phase of the trial, agreed with an independent statistician, and made publicly available via the VeRMiS website, prior to final database lock.

The primary analysis of the primary outcome will be undertaken in line with a modified Intention-to-Treat (ITT) principle amongst participants with complete data at baseline and 26 weeks. The primary analysis will utilise analysis of covariance (ANCOVA), with robust standard errors if necessary, comparing DHI at 26 weeks between the two allocated groups, adjusted for baseline DHI scores, as well as the minimisation factors described previously. Between-group mean differences will be presented with 95% confidence intervals wherever possible, and hypothesis testing will be undertaken at the 5% significance level.

Sensitivity analyses of the primary outcome will be undertaken, possibly including but not limited to per protocol or complier-average causal effect (CACE) analyses. A CACE analysis can provide an unbiased estimate of the intervention effect based on those who complied with their allocated group’s protocol. Additional adjustments in the ANCOVA model will be considered, if any notable imbalances between groups at baseline are observed, in order to investigate the robustness of the conclusions of the primary analyses.

Secondary outcomes will also be analysed using ANCOVA and exploring variable transformations where appropriate.

#### Exploratory and subgroup analyses

- Exploratory analyses will be considered which will help to inform future studies. These include:
  - Exploration of the impact of the following covariates on changes with treatment:
    diagnosis (central versus peripheral versus combined vestibulopathy)
    visual dependency as determined by the rod and disc test
    psychological state as determined by the HADS
    associated symptoms (knee extensor strength, reaction time, leg sensation, neck range of motion)
  - Estimating the diagnostic accuracy of the bedside tests for vestibulopathy

The sensitivity and specificity of diagnosing central and peripheral vestibular disorders using the bedside tests for vestibulopathy compared to laboratory-based measures will be calculated and presented with 95% confidence intervals for the estimates. This is an exploratory analysis that will inform future studies. The relationship between the diagnosis according to clinical tests and recovery with rehabilitation will be explored using an additional subgroup analysis.

### Economic evaluation

The economic evaluation will establish the resources required to provide the customised and booklet based VR, estimate the cost of the interventions, and conduct a full cost-effectiveness analysis (CEA). The intervention costing and CEA, based on within-trial data collection, will be undertaken from the primary perspective of NHS/Social Care. Participant and broader societal perspectives will be considered in sensitivity analyses. The CEA will synthesise cost and outcome data to present incremental cost-effectiveness ratios (ICERs) i.e. the incremental cost per unit of additional outcome. The CEA will present an ICER for the primary outcome (Dizziness Handicap Inventory) and the primary economic endpoint of policy relevance (the QALY). Cost-effectiveness acceptability curves will be presented, as appropriate.

Intervention resource requirements (e.g. physiotherapist time, travel, telephone calls, documentation etc.) and costs will be estimated by case report forms. Self-reported health and social care resource use data will be collected at baseline, 14 weeks (+/− 2 weeks) and 26 weeks (+/− 2 weeks) [90]. This will include primary, secondary and social care, and participant and carer-related resource use data. The EQ-5D-5L will be used to estimate QALYs for use as the primary economic endpoint. Incremental costs and incremental QALYs over 3 months will be used to estimate the cost-per-QALY for customised VR versus the generic booklet-based exercise. A sensitivity analysis will also explore the cost-effectiveness of the intervention when the MSIS-8D or MSIS-8D-P is used as a condition-specific QALY alternative to the EQ-5D. Descriptive statistics will summarise the costs (by type of service) and QALYs. A regression model will be used to adjust for systematic differences between intervention and control arms that have not been accounted for by randomisation. If appropriate, multiple imputation will be used to correct for bias that may result from data that is missing at random, e.g. EQ-5D-5L or cost data [91]. Analyses will also explore uncertainty, and provide a clear, policy-relevant presentation of findings.

### Nested observational study

Treatment plan: Following the diagnosis of BPPV at the initial screening assessment an Epley or Semont manoeuvre will be performed for people identified as having a posterior canal BPPV [57]. The type of manoeuvre chosen will be recorded and determined by factors such as the ability of the participant to move and range of motion and pain in the neck. Participants diagnosed with horizontal canal BPPV will have treatment with the “barbecue rotation” manoeuvre [92] or Forced Prolonged Position [93]. After the manoeuvre BPPV will be re-tested. Up to two manoeuvres will be performed.

Participants will be asked to return to the clinic after 1 week to re-test for any residual BPPV (session 2. The appropriate test for BPPV will be repeated. If the test for BPPV is negative (the primary outcome measure) and participants are symptom free (based on a Vertigo Symptom Scale score < 0.3/4) they will be re-tested on the secondary outcome measures (questionnaires and Dynamic Gait Index DGI). If the test for BPPV is positive the appropriate manoeuvre will be repeated (Eply/Semont or barbecue rotation). After the manoeuvre BPPV will be re-tested. Up to two manoeuvres will be performed.

Participants who still have a positive diagnosis of BPPV in session 2 will be asked to return again to clinic 1 week later. People will be tested for BPPV (the primary outcome measure) and the secondary outcome measures (questionnaires and DGI). If the test for BPPV is negative (the primary outcome measure) and participants are symptom free (based on a Vertigo Symptom Scale score < 0.3/4) no further action will be taken. If the test for BPPV is positive or if people have ongoing vertigo and/or balance and gait impairment they will be provided with a self-management “Balance rehabilitation” booklet providing comprehensive advice on VR exercises. The number of treatment sessions and manoeuvres performed will be recorded.

#### Data measurement

Primary and secondary outcomes will be tested at the end of the treatment session (session 2 or 3) and at 26 and 52 weeks (+/− 2 weeks) after their last treatment appointment.

The primary outcome measure is the clinical test for BPPV (Dix- Hallpike) and (horizontal head rotation with the head in 20^o^ flexion).

Secondary outcome measures will be subjective vestibular specific questionnaires (Dizziness Handicap Inventory [68], MS-12 item self-report walking scale Version 2.0 [81]; Activities-Specific Balance Confidence Scale [82]; Vertigo Symptom Scale- Short Form [59, 83]; Situational Characteristic Questionnaire [52]; Fatigue Scale for Motor and Cognitive functions (FSMC) [84]; Hospital Anxiety and Depression Scale (HADS) [85]; 29-item Multiple Sclerosis Impact Scale [MSIS-29] Version 2.0 [86, 87]) and the Dynamic Gait Index [68, 94].

Participants will be asked to record re-occurrence of their symptoms on a re-occurrence/ AE form during this time if they experience vertigo again so the time of re-occurrence can be noted. If symptoms reoccur participants will be referred to their GP.

#### Analysis

The analyses for observational study will take cognisance of the STROBE guidance [95]. The primary interest in the observational study is on the BPPV reoccurrence rate. The success rate following treatment for BPPV will be estimated and presented with 95% confidence intervals.

The short term (1 week) and longer term (6 and 12 months) reoccurrence rates in people with MS will be estimated and presented with 95% confidence intervals. These rates will be compared to standardised effect sizes from previous literature monitoring BPPV treatment outcomes in people with BPPV and without any additional pathology.

### Data management, audit and monitoring

Data will be recorded on study specific CRFs by the research therapists. Study data will be managed using REDCap electronic data capture tools hosted by PenCTU. Double data entry will be undertaken and discrepancies will be clarified using the original paper CRFs.

Participants’ anonymity will be maintained on all research documents. Data will be collected and stored at the University of Plymouth for a minimum of 10 years in accordance with the Data Protection Act 1988 and GDPR regulations 2018 and will be accessible for the purposes of monitoring, auditing, or at the request of the regulatory agency.

### Trial management and oversight committees

Two committees are involved in trial setup, management and oversight: The Trial Management Group (TMG) and the Trial Steering Committee (TSC). The TMG consists of members of the research team with representation from the CTU and the trial Sponsor. The TMG meets approximately monthly and oversees the general management of the trial and release of the trial results and publications. The TSC consists of members who are mainly independent of the sponsors and investigators (chair, external statistician and lay member). The TSC meets approximately every 6 months and will monitor reports of adverse events, recruitment and attrition rates, the project timeline and finances. It has the ability to see unblinded adverse event data if it is perceived that the frequency of events is higher than anticipated and advise on trial progression.

### Dissemination plan

On completion of the trial, the full study report will be accessible on the study website page (https://www.plymouth.ac.uk/research/vermis), as will the trial protocol and statistical analysis plan. The protocol has been written and published in line with SPIRIT guidelines [96]. Publications will follow CONSORT guidance [97], including the extension for Patient Reported Outcomes (CONSORT PRO) [98], and the template for intervention description and replication (TiDIER) guidelines [99]. Consolidated Health Economic Evaluation Reporting Standards (CHEERS) guidelines for reporting cost-effectiveness studies will be followed [100]. Authorship of the intended articles will be the study team; professional writers will not be used. Dissemination will target users, clinicians and researchers. Results will be presented at national and international conferences and through MS organisation newsletters and talks to local and national support groups. If the customised VR programme is shown to be superior to generic VR rehabilitation, the training materials (treatment manual, training videos on assessment and treatment) will be made available via the study website. We would also aim, in close collaboration with the UK MS Society, to implement training programmes to teach healthcare professionals who regularly see people with MS on the techniques required for diagnosis and treatment. All participants, with their consent, will receive notifications of trial progress and outcomes through study-specific newsletters.

## Discussion

Preliminary evidence suggests that people with MS may benefit from vestibular rehabilitation [38, 39]. However, in previous trials the exact cause of any dizziness has not been defined. This is important as recent work suggests that a substantial proportion of people with MS have BPPV which requires management through different techniques to VR [22, 23]. The overall design of the current study allows people with a discrete diagnosis of peripheral or central vestibulopathy to be identified for the RCT whilst providing appropriate treatment for other causes of vertigo and/or dizziness (i.e. BPPV). Within the United Kingdom, people with MS are not routinely seen by neuro-otologists despite the relatively high incidence of vestibular-symptoms; therefore, this study design overcomes potential ethical issues over not providing timely treatment for BPPV identified through screening for the RCT.

The study employs comprehensive screening involving clinical and laboratory testing of vestibular function to define the cause of the symptoms. In people with MS with defined vestibulopathy, the prognosis for treatment with VR may depend on the person’s presentation such as the cause of any central vestibulopathy and the presence of additional sensorimotor, cognitive and affective impairments. These could affect the recovery process, vestibular compensation, and the ability to undertake VR [27, 101]. Type of vestibulopathy (central or peripheral) and severity of presentation (defined according to the DHI) are therefore minimisation factors in the randomisation process. Fampridine (Dalframpridine, 4-aminopyridine) blocks K^+^ channels that become exposed during the demyelination process. It improves walking in ~ 39% of people with MS. [102] It is also used in the treatment of episodic ataxia [103] with some preliminary evidence suggesting it may improve symptoms of ataxia in people with hereditary cerebellar ataxia [104]. In people with MS, fampridine has been shown in case reports to additionally improve internuclear opthalmoplegia [105] and upper limb ataxia [106] although side effects of dizziness have been reported [107–109]. Therefore, as fampridine may have an effect on the central vestibular pathways and their connections to / from the cerebellum in people with MS, it is also included as a factor in the minimszation algorithm.

This study uses laboratory-based measures to aid with the diagnosis of the cause of vestibulopathy. However, access to these tests may not be readily available within the wider healthcare system to diagnose people with MS. As described above, understanding the exact cause of vestibulopathy may impact on prognosis and therefore management strategies. Therefore, bedside tests for vestibulopathy will also be taken and their sensitivity and specificity and ability to predict recovery with the rehabilitation interventions explored. This will provide preliminary explorative data as to applicability of such tests instead of laboratory-based measures to clinically screen and to aid implementation of findings in the wider healthcare setting.

Exclusion criteria include the use of vestibular sedatives because their use is discouraged in clinical practice for chronic vestibular conditions as it is felt to interfere with the vestibular compensation process [28]. Some anti-emetics, such as prochlorperazine, can also decrease migraine but following chronic (> 1 month) use of this medication there can be a rebound migraine when it is stopped [110]. Therefore, people who have been taking vestibular sedatives for more than 1 month will be excluded. However, if with approval of their neurologist and/or GP, they stop the medication then they will be eligible to take part in the trial after a 6 week wash out period.

Migraine can involve the vestibular system resulting in prodromal symptoms such as vertigo [111]. Although there are preliminary trials suggesting that vestibular migraine can be managed with VR there are other interventions such as lifestyle strategies, reduction in triggers and pharmacotherapy that need to be considered [112, 113]. This, and the differences in presentation and potential prognosis, mean that people with severe migraine (with or without overt vestibular signs) are excluded from the trial. The number of people excluded for this reason will be monitored and reported on the CONSORT diagram. People with less severe migraine will be included in the trial if they have signs of vestibulopathy. Based on clinical experience, a lower number of exercises will be initially provided (three compared to five exercises) to avoid potential migraine exacerbation.

## Trial status

Recruitment started in January 2019 and is ongoing.

## Supplementary Information

**Additional file 1.** Interview Schedule.

## Data Availability

Anonymised data generated as a result of this study will be made available publically via the VeRMiS website (https://www.plymouth.ac.uk/research/vermis) approximately 1 year after publication of the final manuscript.

## Abbreviations

ABC: Activities specific balance confidence scale
BPPV: Benign paroxysmal position vertigo
CONSORT: Consolidated standards of reporting trials
CRF: Case report form
DGI: Dynamic gait index
DHI: Dizziness handicap inventory
EDSS: Expanded disability status scale
FSMC: Fatigue scale for motor and cognitive function
HRA: Health research authority
HC-BPPV: Horizontal canal BPPV
HADS: Hospital anxiety and depression scale
ISRCTN: International standard randomised controlled trials number
MS: Multiple sclerosis
MSIS-29vs2.0: MS impact scale (29 item) version 2
MSWS-12vs2.0: MS walking scale (12 item) version 2
OKR: Optokinetic reflex
SDMT: Symbol digit modalities test
SCV: Slow component velocity
VR: Vestibular rehabilitation
VSS: Vertigo symptom scale
VOR: Vestibulo-ocular reflex
