# Supplementary material for: Vestibular rehabilitation in multiple sclerosis: study protocol for a randomised controlled trial and cost-effectiveness analysis comparing customised with booklet based vestibular rehabilitation for vestibulopathy and a 12 month observational cohort study of the symptom reduction and recurrence rate following treatment for benign paroxysmal positional vertigo
Source: BMC Neurol. 2020 Nov 27;20:430. doi: 10.1186/s12883-020-01983-y (PMC7694922; doi:10.1186/s12883-020-01983-y)
Supplement: Supplementary file 1 — Additional file 1. Interview Schedule. [file 12883_2020_1983_MOESM1_ESM.docx]

Title: Vestibular Rehabilitation in Multiple Sclerosis: Study protocol for a randomised controlled trial and cost-effectiveness analysis comparing customised with booklet based vestibular rehabilitation for vestibulopathy and a 12 month observational cohort study of the symptom reduction and recurrence rate following treatment for Benign Paroxysmal Positional Vertigo.

**Interview Schedule**

*Thank you very much for taking part in this study. <Introduce name, place of work>.* I would like to ask some questions about your experience with the study and undertaking the exercises prescribed. This will help us when teaching healthcare professionals and people with MS the best way to undertake the exercises and in developing future exercise studies.

The interview is very informal and completely confidential. Only my colleagues and I will see your responses and your name will not appear in anything we write. With your permission I would like to record the interview. This is so that I can concentrate on what you are telling me rather than spending the whole time taking notes. Is that okay.?

As soon as we have written up your interview and anonymised it, the recording will be destroyed.

*I would like to start off by asking some general questions about the study*

1. Opinion on effect of exercises and group allocation?

| Main Question | Possible Probes |
| --- | --- |
| Which group were you in – did you use the booklet based exercises or have exercises that were monitored and progressed by a therapist on a weekly basis?  Did you feel any change with the exercises?  Were you happy with the group you were allocated to? | If so what changed?  If not would you prefer to be in the other group / what would you have preferred? |

2. Opinion on exercise programme structure?

| Main Question | Possible Probes |
| --- | --- |
| What did you think about the daily length of the exercises?  What did you think about the content of the exercises?  What did you think about the information provided about the exercises?  Did you find completing the diary useful?  Was the follow up contact with the therapist helpful? | Were there some aspects you liked/did not like?  In the initial training session with the therapist?  In the home exercise programme?  Was the contact face to face /telephone/ e mail. Would you have preferred another type of contact?  Would you have preferred more or less time with the therapist? |

3. Exercise compliance / barriers and facilitators

| Main Question | Possible Probes |
| --- | --- |
| Were you able to complete the exercises every day?  Did you find anything that helped you undertake the exercises?  Were there any factors hindered / stopped you from completing the exercises?  Would you consider carrying on with the exercises in the future?  Are there any other suggestions you feel that would help you undertake the exercises? | Did you find any solutions to the factors that hindered you completing the exercises?  If not why would you not carry on  What frequency of exercise would you consider suitable |

< Thank participant and ask if there is anything else they would like to add>
